# Unravelling the Therapeutic Potential of Botanicals Against Chronic Obstructive Pulmonary Disease (COPD): Molecular Insights and Future Perspectives

**DOI:** 10.3389/fphar.2022.824132

**Published:** 2022-05-11

**Authors:** Sicon Mitra, Uttpal Anand, Mimosa Ghorai, Balachandar Vellingiri, Niraj Kumar Jha, Tapan Behl, Manoj Kumar, Radha, Mahipal S. Shekhawat, Jarosław Proćków, Abhijit Dey

**Affiliations:** ^1^ Department of Biotechnology, School of Engineering and Technology, Sharda University, Greater Noida, India; ^2^ CytoGene Research & Development LLP, Lucknow, Uttar Pradesh, India; ^3^ Department of Life Sciences, Presidency University, Kolkata, India; ^4^ Human Molecular Cytogenetics and Stem Cell Laboratory, Department of Human Genetics and Molecular Biology, Bharathiar University, Coimbatore, India; ^5^ Department of Pharmacology, Chitkara College of Pharmacy, Chitkara University, Chandigarh, India; ^6^ Chemical and Biochemical Processing Division, ICAR-Central Institute for Research on Cotton Technology, Mumbai, India; ^7^ School of Biological and Environmental Sciences, Shoolini University of Biotechnology and Management Sciences, Solan, India; ^8^ Department of Plant Biology and Biotechnology, Kanchi Mamunivar Government Institute for Postgraduate Studies and Research, Puducherry, India; ^9^ Department of Plant Biology, Institute of Environmental Biology, Wrocław University of Environmental and Life Sciences, Wrocław, Poland

**Keywords:** lungs, inflammation, alternative therapy, medicinal plants, COVID-19, COPD, clinical efficacy, plant-based formulation

## Abstract

**Background:** COPD (chronic obstructive pulmonary disease) is a serious health problem worldwide. Present treatments are insufficient and have severe side effects. There is a critical shortage of possible alternative treatments. Medicinal herbs are the most traditional and widely used therapy for treating a wide range of human illnesses around the world. In several countries, different plants are used to treat COPD.

**Purpose:** In this review, we have discussed several known cellular and molecular components implicated in COPD and how plant-derived chemicals might modulate them.

**Methods:** We have discussed how COVID-19 is associated with COPD mortality and severity along with the phytochemical roles of a few plants in the treatment of COPD. In addition, two tables have been included; the first summarizes different plants used for the treatment of COPD, and the second table consists of different kinds of phytochemicals extracted from plants, which are used to inhibit inflammation in the lungs.

**Conclusion:** Various plants have been found to have medicinal properties against COPD. Many plant extracts and components may be used as novel disease-modifying drugs for lung inflammatory diseases.

## Graphical Abstract

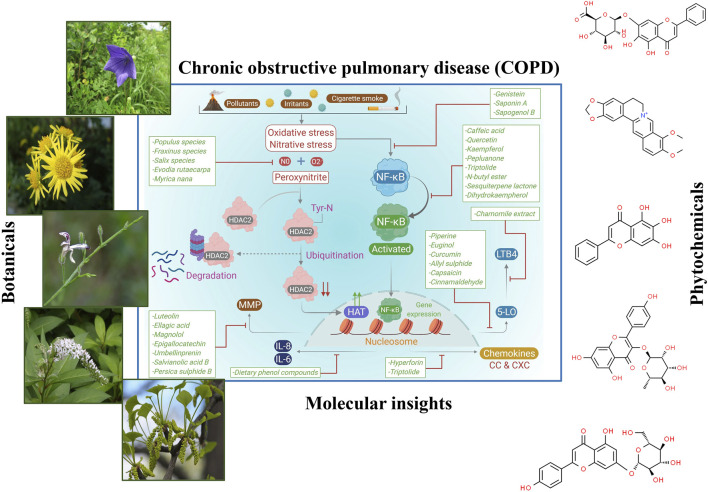

## 1 Introduction

Chronic obstructive pulmonary disease (COPD) is characterized by airflow restriction that does not completely reverse and is a significant cause of morbidity and death globally (GBD 2015 Chronic Respiratory Disease Collaborators, 2017; Vogelmeier et al., 2017; Anzueto and Miravitlles, 2018). Both asthma and COPD are the most common respiratory illnesses worldwide that are characterized by airway blockage and persistent respiratory inflammation. However, the pattern is noticeably different from one another. In the case of asthma, inflammation begins with CD4^+^ T helper 2 (Th2) cells, as well as dendritic cells. This further proceeds by eosinophilic infiltration along with sensitization of the mast cell. This results in the release of many inflammatory mediators. However, COPD is marked by an increase in the number of neutrophils and T lymphocytes in the lungs, leading to a significant increase in activated macrophages (Barnes, 2008).

Tobacco use is a key risk factor for COPD, yet despite decades of lowering smoking rates in many countries, associated reductions in disease burden have been modest (Adeloye et al., 2015). In population-based observational samples from 1987 to 1988 and 2005 to 2009, only a small percentage of lifetime smokers were found to have spirometry-defined COPD with up to 30% occurring among people who had never smoked (Bakke et al., 1991). Standard medicines are ineffective and have a slew of negative side effects. As a result, there has been a strong push for safer and potentially effective alternative treatments. Medicinal plants are the oldest and most widely used for treating a variety of human diseases (Anand et al., 2019; Anand et al., 2020; Anand et al., 2021). Traditional medicine and ethnobotany had always played crucial roles in reducing human morbidity and mortality (Biswas et al., 2021; Dutta et al., 2021; Paul et al., 2021). Crude plant extracts and preparations have been recommended against a variety of human ailments (Das et al., 2021; Mohammed et al., 2021; Tandon et al., 2021). A number of phytoconstituents have also been reported as promising disease modifying agents (Bandopadhyay et al., 2021; Banerjee et al., 2021; Datta et al., 2021). Many investigations have also carried out to explore the pre-clinical and clinical efficacy of botanical-derived products (Khare et al., 2021; Mitra et al., 2021; Mitra et al., 2022b). However, COPD could be treatable if exposure to risk factors can be avoided (Vogelmeier et al., 2020). In terms of COPD, several plants have been suggested in many nations that may be effective (Zhou et al., 2016; Hwang and Ho, 2018; Sun et al., 2020). However, only the bare minimum of solid scientific evidence is accessible in the literature. Except for a few early research works where detailed examinations of any plant or its derived compounds have not been conducted specifically for COPD patients. In this review, we have included several plants that have been highlighted for their effectiveness in patients with COPD.

## 2 Databases and Search Strings Used to Retrieve Literature

Google Scholar (https://scholar.google.com/) search engine was given the most attention in this review article since it provides a straightforward approach to search for various scholarly publications. As a result, this was utilized as an index to a wide range of scientific publications. Furthermore, additional journal articles available on the internet helped to find this literature review study. This includes databases used in particular for retrieving published research, such as ScienceDirect, Elsevier, PubMed and Scopus. The relevant literature was recovered using search -strings like “COPD,” “COVID-19,” “coronaviruses,” “inflammation,” “medicinal plants,” and “conventional therapeutics” in various combinations. The retrieved liretaure and cross-referencing among them included the references describing the potential of plant and plant-derived phytochemicals against COPD, are dicussed in the present artcle.

## 3 Epidemiology

Due to the paucity of data representative of the worldwide population and the lack of agreement on case definitions, studying the global prevalence of COPD was previously challenging. However, the scope and start of international COPD research have increased our awareness of the disease’s worldwide impact and shown the prevalence of varying diseases across nations. Global burden of disease (GBD) research employed national surveys, census data, and a central database of registries from more than 100 nations, stratified by sociodemographic index (SDI), a composite measure of fertility, income, and education (GBD Chronic Respiratory Disease Collaborators, 2017). According to a comprehensive review of population-based research conducted in 52 countries in 2015, the Americas had the highest prevalence of COPD (15% in 2010), while Southeast Asia had the lowest (10%). The study predicted a global prevalence of 12%, equivalent to 384 million cases in 2010, and a figure far higher than the GBD study estimate (Adeloye et al., 2015).

## 4 Molecular Events Involved in COPD

From the study by Osoata et al. (2009), it was found that HDAC2 expression was decreased by nitration of certain tyrosine residues under nitrative/oxidative stress. *In vitro*, hydrogen peroxide, peroxynitrite, and cigarette smoke-conditioned media decreased HDAC2 expression in A549 epithelial cells. This decrease was caused by enhanced proteasomal degradation followed by ubiquitination and did not decrease mRNA production or stability. HDAC2 was nitrated in the peripheral lung tissues of smokers and patients with COPD, as well as under nitrative/oxidative stress (Osoata et al., 2009). Furthermore, oxidative stress has been implicated in the decrease in sirtuin-1 (SIRT1), which is a crucial anti-aging molecule that is both a protein deacetylase and plays a key role in controlling MMP-9 (Stockley et al., 2009). Reactive oxygen species (ROS) are also important in the development of COPD. Tobacco smoke includes significant levels of oxidants and generates a wide range of free radicals, including ROS. Excess ROS production causes oxidative stress, increasing inflammatory responses and leading to the severe stage of COPD. Nuclear factor erythroid 2-related factor 2 (Nrf2) is induced via a Keap1-dependent signaling mechanism in which Nrf2 is inhibited at the basal level via Keap1-controlled ubiquitination-proteasomal degradation. It is induced by oxidants and electrophiles via alteration of critical cysteine thiols in Keap1 and Nrf2. Activated Nrf2 regulates drug metabolism, antioxidant defense, and oxidant signaling by mediating the increased production of a slew of enzymes and signaling proteins, regulating oxidant physiology and disease. Many plants constituents such as eriodictyol, baicalein etc. are therefore found to regulate the Nrf2 pathway against COPD (Ma, 2013). Myeloperoxidase is most known for its capacity to catalyze reactive oxidants, which aid in the elimination of infections. Oxidants generated by myeloperoxidase leads to tissue damage as well as the development and spread of acute and chronic vascular inflammation. Myeloperoxidase from neutrophils also plays a crucial role in cancer growth and progression (Valadez-Cosmes et al., 2021). Plant constituents like fisetin, morin, etc. tend to regulate myeloperoxidase. As a result, numerous oxidative stress-related molecules, such as NADPH oxidase, Nrf2, superoxide dismutase, and myeloperoxidase may be considered as the potential targets for COPD treatment. PI3K-mediated signaling in neutrophils and macrophages is involved in inflammation and immunological responses, and its activity is increased in the lungs of COPD. In a mouse smoke model, inhibition of certain PI3K isoforms decreased lung neutrophilia (Doukas et al., 2009).

Several literary works have so far identified that PI3K inhibitors are indispensable for potentialand effective COPD treatments. Furthermore, inhibitors targeting nuclear transcription factor-B (NF-κB), which plays a key role in the encoding of numerous inflammatory genes and related kinases such as IκB kinase, have been explored (Schuliga, 2015). From the studies of Renda et al. (2008), it has been reported that the presence of active p38 MAPK in alveolar spaces and alveolar walls of smokers with COPD suggests that activation of the MAPK pathway is a critical stage in the disease aetiology. Western blot examination verified the enhanced expression of phosphorylated p38 in COPD patient’ alveolar macrophages. Moreover, the expression of phospho-p38 was linked to deterioration of lung function and the amount of CD8 T-lymphocytes invading the walls of alveoli. Therefore, p38-MAPK can be used as a potential molecular target for the synthesis of novel and more effective drugs for the treatment of COPD. In the smoke-induced mouse model system, NF-κB can be inhibited by intratracheal administration of NF-κB decoy oligodeoxynucleotides (ODNs), and decoy ODN-mediated NF-κB inhibition can suppress smoke-induced lung inflammation, respiratory dysfunction, and improve pathological changes in the lung parenchyma (Renda et al., 2008).

Pro-inflammatory cytokines including interleukin-1 (IL-1), tumor necrosis factor- α (TNF-α), and IL-6, as well as chemokines such as IL-8 are vital in the pathogenic system because they can induce and recruit circulating cells. The transforming growth factor-β (TGF-β) has been linked to airway fibrosis, which can result in airway damage (Rovina et al., 2009). Therefore, in therapeutic trials treating COPD, many techniques for inhibiting such cytokines or their receptors were explored. Hesperidin inhibited the synthesis of pro-inflammatory cytokines such as IL-6 and TNF-α while increasing the synthesis of anti-inflammatory cytokines like IL-10 and IL-4. Hesperidin activity may be regulated via the disruption of the AP-1 and NF-κB pathways (Yeh et al., 2007).

## 5 COVID-19 is Associated With Mortality and the Severity of COPD

According to the studies by Alqahtani et al. (2020), the frequency of COPD in patients with COVID-19 was low. However, mortality (60%) and the risk of severity (63%) were high, implying that COPD patients with positive COVID-19 infection are at increased risk of major complications and even death. Furthermore, the proportion of current smokers with COVID-19 infection was 9% [95% confidence intervals (CI), 4–14%] and this was associated with higher severity (22.30%) and death (38.5%) (Alqahtani et al., 2020). Although the incidence of COPD with the verified COVID-19 cases was not great, however, COVID-19 imposes a significant burden on patients with COPD with increasing disease severity (Zhang et al., 2020a; Guan et al., 2020). Furthermore, data from two investigations on COPD patients suffering from COVID-19 infection reveal a mortality rate of 60% (Zhang et al., 2020b; Yang et al., 2020). Despite the fact that COPD is not very common in reported cases of COVID-19, COVID-19 infection is associated with substantial severity and death in COPD. Current smokers were also at increased risk of serious illness and death. To minimize the risk of COVID-19 in COPD patients and current smokers, effective preventive interventions are urgently needed.

## 6 Adverse Effects of Conventional Therapies for the Treatment of COPD

Several adverse effects are associated with conventional therapy of COPD. There exists one type of treatment for COPD, which is symptomatic pharmacological and based on bronchodilators, i.e., glucocorticoid, β2-adrenoreceptor agonists, theophylline, anticholinergics, and a combination of such drugs (Montuschi, 2006). However, in the case of β2-adrenoreceptor agonists, there are several adverse effects, such as myocardial ischemia, electrolyte imbalance, tachycardia, hypertension, osteoporosis etc. Due to such adverse effects, this group of drugs is not recommended for the treatment of COPD. Anticholinergic drugs impart many adverse effects such as blurred vision, cognitive disorders, constipation, urinary complications, and dryness of mouth when used for COPD treatment. Similarly, glucocorticoids are avoided because of their high cost, side effects, and high-risk factor. Alongside, theophylline also has many notable side effects, *viz.* headache, vomiting, diarrhoea, myocardial infraction, nausea, arrhythmias, and restlessness (Gupta et al., 2008). Side effects of steroids include blurred vision, hypertension, increased appetite, glaucoma, and weight gain (Hubbard and Tattersfield, 2004). Furthermore, the use of steroids for the treatment of COPD can negatively affect innate immunity and leads to susceptibility to many other diseases. Therefore, the development of new alternative therapeutics with safer pharmacological approaches must be introduced for the treatment of COPD (Sing and Loke, 2010).

## 7 Medicinal Plants Used for the Treatment of COPD

**FIGURE 1:**
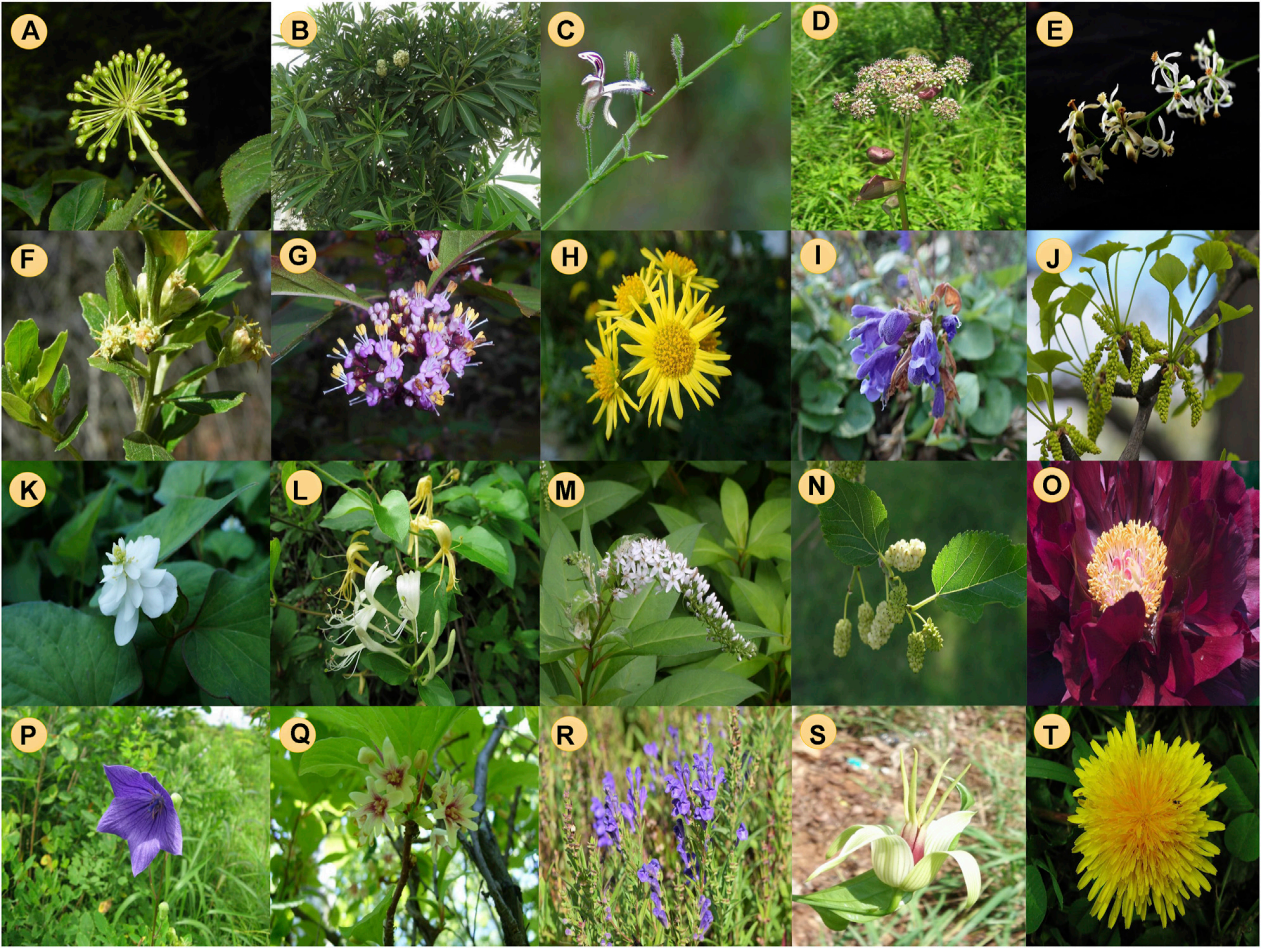
Medicinal plants investigated against COPD. **(A)** Eleutherococcus senticosus (= Acanthopanax senticosus), **(B)** Alstonia scholaris, **(C)** Andrographis paniculata, **(D)** Angelica decursiva, **(E)** Azadirachta indica, **(F)** Baccharis retusa, **(G)** Callicarpa japonica, **(H)** Chrysanthemum indicum, **(I)** Dracocephalum rupestre, **(J)** Ginkgo biloba, **(K)** Houttuynia cordata, **(L)** Lonicera japonica, **(M)** Lysimachia clethroides, **(N)** Morus alba, **(O)** Paeonia × suffruticosa, **(P)** Platycodon grandiflorum, **(Q)** Schisandra chinensis, **(R)** Scutellaria baicalensis, **(S)** Stemona tuberosa, **(T)** Taraxacum campylodes (= T. officinale).

**FIGURE 2:**
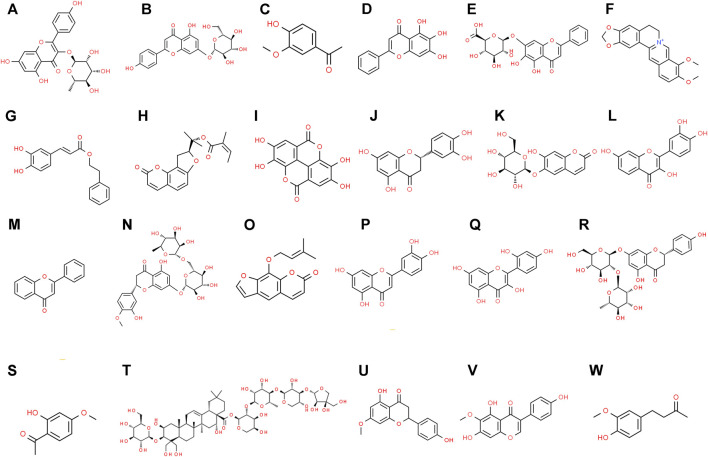
Chemical structures of natural plant products reported against COPD. **(A)**. Afzelin, **(B)**. Apigenin-7-glucoside, **(C)**. Apocynin, **(D)**. Baicalein, **(E)**. Baicalin, **(F)**. Berberine, **(G)**. Caffeic acid phenethyl ester, **(H)**. Columbianadin, **(I)**. Ellagic acid, **(J)**. Eriodictyol, **(K)**. Esculin, **(L)** Fisetin, **(M)** Flavone, **(N)** Hesperidin, **(O)** Imperatorin, **(P)** Luteolin, **(Q)** Morin, **(R)** Naringin, **(S)** Paeonol, **(T)** Platycodin D, **(U)** Sakuranetin, **(V)** Tectorigenin, **(W)** Zingerone.

Over the past few years, many medicinal plants have been extensively studied for their properties against COPD. After a thorough investigation of such medicinal plants, they have been considered as an alternative treatment source to the systemic treatment of different types of lung-related diseases. In this review, the different plants conferring such medicinal properties are listed in Table 1. Some of the brief reports regarding those medicinal plants are also described below. Figure 1 presents the medicinal plants investigated against COPD. Figure 2 represents the natural chemical structures of the plant natural products reported against COPD.

**TABLE 1:** Different plant extracts inhibiting lung inflammation.

Sl no.	Plants	Inflammagen used	Extracts	Reference
1.	Aconitum tanguticum (Maxim.) Stapf [Ranunculaceae]	LPS (rat)	Alkaloid fraction	Wu et al. (2014a)
2.	Alisma plantago-aquatica subsp. orientale (Sam.) Sam. (= Alisma orientale (Sam.) Juz.) [Alismataceae]	LPS	80% ethanol	Kim et al. (2013)
3.	Alstonia scholaris (L.) R. Br. [Apocynaceae]	LPS (i.t.) (rat)	Alkaloid fraction	Zhao et al. (2016)
4.	Angelica decursiva (Miq.) Franch. and Sav. [Apiaceae]	LPS	70% ethanol	Lim et al. (2014)
5.	Asparagus cochinchinensis (Lour.) Merr. [Asparagaceae]	LPS	70% ethanol	Lee et al. (2015a)
6.	Azadirachta indica A. Juss. [Meliaceae]	Cigarette smoke	Water	Koul et al. (2012)
7.	Callicarpa japonica Thunb. [Lamiaceae]	Cigarette smoke	Methanol	Lee et al. (2015b)
8.	Canarium lyi C.D. Dai & Yakovlev [Burseraceae]	LPS	Methanol	Hong et al. (2015)
9.	Chrysanthemum indicum L. [Asteraceae]	LPS (i.t.)	Supercritical CO2 extract	Wu et al. (2014b)
10.	Cnidium monnieri (L.) Cusson [Apiaceae]	Cigarette smoke extract/LPS (i.t.)	Water	Kwak and Lim (2014)
11.	Eleutherococcus senticosus (Rupr. and Maxim.) Maxim. (= Acanthopanax senticosus (Rupr. and Maxim.) Harms) [Araliaceae]	LPS (i.t.)	-	Fei et al. (2014)
12.	Ginkgo biloba L. [Ginkgoaceae]	LPS (i.t.)	Egb761	Huang et al. (2013)
13.	Houttuynia cordata Thunb. [Saururaceae]	LPS	70% ethanol	Lee et al. (2015c)
14.	Isodon japonicus var. glaucocalyx (Maxim.) H.W.Li (= Rabdosia japonica var. glaucocalyx (Maxim.) H.Hara) [Lamiaceae]	LPS (i.t.)	Flavonoid fraction	Chu et al. (2014)
15.	Lonicera japonica Thunb. [Caprifoliaceae]	LPS (i.t.)	50% ethanol	Kao et al. (2015)
16.	Lysimachia clethroides Duby [Primulaceae]	LPS	Methanol	Shim et al. (2013)
17.	Morus alba L. [Moraceae]	LPS	70% ethanol	Lim et al. (2013)
18.	Schisandra chinensis (Turcz.) Baill. [Schisandraceae]	LPS	Water	Bae et al. (2012)
19.		Cigarette smoke-induced	Aqueous ethanol	Zhong et al. (2015)
20.	Stemona tuberosa Lour. [Stemonaceae]	Cigarette smoke	Water	Lee et al. (2014)
21.	Taraxacum mongolicum Hand.-Mazz. [Asteraceae]	LPS	Water	Ma et al. (2014)
22.	Tripterygium wilfordii Hook. f. [Celastraceae]	LPS	Ethanol	Brinker et al. (2007)

[Figures are obtained from Wikimedia Commons under the Creative Commons Attribution-Share Alike 4.0 license (a, b, c, e, g, h, i, j, k, p, r, s); Creative Commons Attribution-Share Alike 3.0 license (d, m); Creative Commons Attribution-Share Alike 2.0 license (f); Creative Commons Zero, Public Domain Dedication (l, o, q, t); Creative Commons Attribution-Share Alike 2.1 Spain license (n)].

[The chemical structures are obtained from the free chemical structure database (www.chemspider.com)].

### 7.1 *Aconitum tanguticum*

Wu et al. (2014a) reported that total alkaloids of *Aconitum tanguticum* (TAA) substantially decreased the lung W/D ratio and increased the value of PaO2 or PaO2/FiO2 in ALI rats at 6, 12, and 24 h after lipopolysaccharide (LPS) challenge. TAA also decreased the overall protein content, as well as the total number of cells, neutrophils, and lymphocytes. Furthermore, TAA reduced MPO activity and reduced histological alterations in the lung. Furthermore, TAA also reduced the concentrations of TNF-α, IL-6, and IL-1b concentrations in bronchoalveolar lavage fluid (BALF) at 6, 12, and 24 h after LPS administration. TAA substantially reduced NF-κB activation in lung tissue (Wu et al., 2014a).

### 7.2 *Alstonia scholaris*

This plant belongs to the family of Apocynaceae. Over hundreds of years, it has been traditionally used for the treatment of respiratory diseases such as asthma, cough, COPD, phlegm, etc. Total alkaloids (TA) isolated from *Alstonia scholaris* leaves were tested for their ability to protect rats against lipopolysaccharide (LPS)-induced airway inflammation (AI), and TA was found to reduce the proportion of WBC, AKP, LDH, and ALB levels, and neutrophils in the BALF while increasing the ALB content in the blood. It also raised nitric oxide (NO) levels in the lungs, serum, and BALF while decreasing MDA concentrations in the lungs. TNF-α and IL-8 production in BALF and lung were also inhibited by total alkaloids. Finally, histological analysis revealed that total alkaloids reduced lung tissue damage in LPS-induced airway inflammation (Zhou et al., 2016).

### 7.3 *Alisma orientale*

LPS-treated mice when treated with ethanol extracts of *Alisma orientale* (EEAO) leads to the suppression of pulmonary inflammation significantly. Septic mice post-treated with EEAO enhanced the survival rate in mice. Therefore, these findings indicate that EEAO has a therapeutic impact on acute lung injury caused by sepsis, implying that EEAO might be used as a therapeutic approach to treat acute lung disorders such as acute lung injury (Kim et al., 2013).

### 7.4 *Angelica decursiva*

Extracts from roots of *Angelica decursiva* demonstrated significant inhibitory action towards LPS-induced lung inflammation in mice. Few coumarin derivatives were identified from the extracts, including columbianadin, umbelliferone, umbelliferone 6-carboxylic acid, nodakenin, and nodakenetin. Among the identified compounds, columbianadin was shown to have potent anti-inflammatory action against IL-1-treated A549 cells and LPS-treated MH-S cells. Columbianadin was discovered to reduce NO synthesis by inhibiting inducible NO synthase. Furthermore, columbianadin was shown to have strong inhibitory action against LPS-induced lung inflammation following oral treatment (Lim et al., 2014).

### 7.5 *Asparagus cochinchinensis*

The ethanol extract of roots from *Asparagus cochinchinensis* (ACE) was reported to prevent IL-6 synthesis from IL-1-treated lung epithelial cells (A549), as well as the primary component, methyl protodioscin (MP), furthermore heavily suppressed synthesis of IL-6, IL-8, and TNF-α from A549 cells. The suppression of c-Jun N-terminal kinase (JNK) and the c-Jun activation pathway was shown to be involved in the downregulation of the synthesis of pro-inflammatory cytokine. In LPS-induced acute lung damage, oral treatment with ACE and MP effectively decreased cell invasion in BALF. In lung parenchyma, methyl protodioscin (MP) also decreased the synthesis of pro-inflammatory cytokines such as IL-6, TNF-α, and IL-1 (Lee et al., 2015a).

### 7.6 *Azadirachta indica*

The modulatory and preventive properties of aqueous *Azadirachtaindica* leaf extract (AAILE) against cigarette smoke-induced pulmonary oxidative stress have been examined. Regular smoking and smoking disrupted the enzymatic and non-enzymatic defense systems of pulmonary tissue, as evidenced by higher levels of MDA, alterations in FTIR spectra, and an increase in the 3H-B [a] P-DNA adduct. AAILE cotreatment, on the other hand, was shown to be protective in terms of these characteristics. As a result, AAILE administration may be useful in combating pro-oxidant conditions caused by cigarette smoke (CS) in the lungs (Koul et al., 2012).

### 7.7 *Callicarpa japonica*

In H292 cells stimulated with cigarette smoke condensate (CSC), *Callicarpa japonica* therapy substantially reduced ERK phosphorylation. There was no discernible reduction in JNK and p38 phosphorylation in response to CSC stimulation. As a result, their data suggest that CJT suppression of MUC5AC synthesis was strongly related to ERK phosphorylation inhibition. CJT reduced neutrophil infiltration and mucus generation in a mouse model with COPD and decreased MUC5AC expression in a CSC stimulated H292 human lung mucoepidermoid cell line (Lee et al., 2015b).

### 7.8 *Gingko biloba*

When administered intraperitoneally, *Ginkgo biloba* leaves extract can significantly inhibited lung inflammation in LPS-induced ALI in modest doses. As a result, this plant material can cure inflammatory or allergic disorders of the lungs. Ginkgolides and flavonoids are the main components present in this plant. Additionally, flavonoid derivatives have anti-inflammatory properties in the lungs (Huang et al., 2013).

### 7.9 *Houttuynia cordata*

In the LPS induced ALI mouse model, *Houttuynia cordata* markedly reduced the synthesis of pro-inflammatory mediators such as IL-6 and NO in lung epithelial cells (A549) and alveolar macrophages (MH-S). Significant flavonoids such as hyperoside, afzelin, and quercitrin were effectively separated from the extract, and they also reduced LPS-induced lung inflammation in mice (Lee et al., 2015c).

### 7.10 *Rabdosia japonica*

The flavonoids fraction of *Rabdosia japonica* var. *glaucocalyx* (RJFs) reduced LPS-induced lung damage by decreasing lung wet-to-dry weight ratio, inhibited protein levels, and increased the synthesis of NO in the BALF. Furthermore, in ALI mice RJF helps in the reduction of TNF-α, IL-6, and IL-1 levels in BALF. Pretreatment of ALI mice by RJF leads to enhancement in the activity of SOD and suppression in the activity of MPO. RJF also leads to dramatically reduced lung damage by lowering complement deposition. Meanwhile, in the serum of ALI mice, RJFs lowered the amount of complement 3. RJF’ anti-ALI actions were linked to suppression of synthesis of pro-inflammatory mediators and a reduction in complement levels (Chu et al., 2014).

### 7.11 *Tripterygium wilfordii*

Triptolide, possibly followed by tripdiolide, is among the most bioactive molecules of *Tripterygium* extract. On the molecular level, a few pharmacological effects of triptolide could be described by the observation that it heavily suppresses transcription of TNF-α and prevents the activation of NF-κB as well as other transcription factors. This further results in the inhibition of transcription of inflammation- and immune-related genes. Triptolide has also been demonstrated to interact with the glucocorticoid receptor. Glucocorticoid-responsive genes cannot be activated by the glucocorticoid receptor-1 complex and may downregulate the expressional activity of NF-κB and AP-1, resulting in a steroid-sparing, and anti-inflammatory effect (Brinker et al., 2007).

## 8 Roles of Other Plants in the Treatment of COPD

In LPS-regulated RAW 264.7 cells, the expression of several pro-inflammatory mediators has been suppressed by *Canarium lyi* and it also inhibited activation of NF-κB and MAPKs in ALI mice (Hong et al., 2015). The extracts of *Chrysanthemum indicum* can successfully reduce LPS-stimulated acute lung injury in mice. The therapeutic efficacy of *C. indicum* was correlated with changes in TLR4 signaling pathways (Wu et al., 2014b). In lung tissues of an ALI mouse AS reduced the levels of IL-6 and TNF-α *via* suppressing the NF-κB pathway (Fei et al., 2014). *Lonicera japonica* has exhibited protective activity against LPS-induced lung inflammatory cytokine release (Kao et al., 2015). When Raw 264.7 cells are pre-treated with Lysimachia clethroides extract, it reduced release of LPS-stimulated NO, and synthesis of interleukin (IL)-1, and IL-6 cells in a dose-dependent manner. LPS-mediated IRF3 and STAT1 phosphorylation was also reduced by the extract (Shim et al., 2013). The ethanol extract of *Morus alba* root barks suppressed bronchitis-like symptoms when examined against LPS-mediated inflammation, as measured by TNF-α production. *M. alba* and its principal flavonoid components, including kuwanone G, norartocarpanone, and kuwanone E reduced synthesis of IL-6 in epithelial cells (A549) of lungs and biosynthesis of NO in lung macrophages (MH-S) (Lim et al., 2013). *Schisandra chinensis* extracts suppressed cytokine mixture-mediated synthesis of NO and lowered secretions of IL-8 and MCP-1 in A549 cells. In LPS-induced BALB/c mice. In addition, the extracts effectively inhibited infiltrations of neutrophil and macrophage infiltrations within lung tissues. Meanwhile, it increased the levels of IL-6 and TNF-α in BALF (Bae et al., 2012). In cough hypersensitive Guinea pigs which are induced by cigarette smoke, *S. chinensis* lowered cough intensity and lung inflammation (Zhong et al., 2015). *Stemona tuberosa* dramatically reduced the number of total cells, lymphocytes, neutrophils, and macrophages in the BALF of mice that are exposed to cigarette smoke. Furthermore, it lowered the levels of cytokines (TNF-α, IL-6) and the tested chemokine (KC) in BALF. Also, it prevented the expansion of the alveolar airways caused by cigarette smoke exposure (Lee et al., 2014). Water extract of *Taraxacum mongolicum* reduced inflammatory cell counts in the BALF, lowered protein levels of PI3K/Akt/mTOR in the lung, enhanced activity of SOD, inhibited the activity of myeloperoxidase, and significantly suppressed LPS-induced neutrophils (Ma et al., 2015).

## 9 Plant Constituents Used in the Treatment of COPD

Some phenolics were also shown to be helpful against pulmonary inflammation when administered orally. Caffeic acid derivative, apocynin, ellagic acid, zingerone, and paeonol are among them. At 10 mg/kg/day, paeonol, the main component of *Paeonia* × *suffruticosa*, reduced “cigarette smoke-induced lung inflammation” in a mouse model of COPD (Liu et al., 2014). This observation is consistent with the ability of the *P.* × *suffruticosa* extract to prevent LPS-induced ALI in rats (Fu et al., 2012). Berberine, one of the active chemicals of *Argemone ochroleuca* [Papaveraceae], was discovered to have a relaxing impact on the tracheal muscle, which might be due to its antagonistic action on muscarinic acetylcholine receptors. Therefore, berberine has been found to be quite effective in the treatment of COPD (Sánchez-Mendoza et al., 2008). Hesperidin, naringin, and sakuranetin were found to function as anti-inflammatory agents in the lungs. In a smoke-induced COPD model, quercetin reduced lung inflammation, and mucus production (Yang et al., 2012). This suppressing effect can be achieved by reducing oxidative stress, decreasing NF-κB activation, and further inhibiting EGFR phosphorylation as some diterpenoids and triterpenoids have been shown to reduce lung inflammation. For example, triterpenoid saponins are important components of the *Hedera helix* [Araliaceae] that often play a key role in the treatment of lung inflammation (Hocaoglu et al., 2012). Platycodin D, which is a triterpenoid saponin, is derived from *Platycodon grandiflorum*, which further inhibited ALI (Tao et al., 2015). Furthermore, in human airway smooth muscle cells (HASMC), salvianolic acid B isolated from *Salvia miltiorrhiza* [Lamiaceae] substantially reduced H_2_O_2_-induced MMP-2 mRNA levels, along with gelatinolytic activity (Zhang and Wang, 2006). MMP activity and MMP-1 expression have been inhibited by umbelliprenin extracted from *Ferula persica* var. *persica* [Apiaceae] and luteolin, which is extracted from *Zostera marina* [Zosteraceae] (Kim et al., 2004; Shahverdi et al., 2006). Polyphenolic substances extracted from the bark of *Tristaniopsis calobuxus* [Myrtaceae], such as epigallocatechin and ellagic acid, reduced the levels of MMP-9 mRNA in mice peritoneal macrophages (Bellosta et al., 2003).

Apigenin-7-glycoside extracted from *Andrographis paniculata* dramatically suppressed LPS-mediated inflammation in the lung, and it also had an anti-inflammatory action *via* the MAPK and NF-κB (IκB) pathways (Li et al., 2015). In hamsters with LPS-enhanced lung injury, treatment with apocynin extracted from *Picrorhiza kurroa*, inhibited the secretion of oxidants from inflammatory cells, as well as apocynin increased the efficacy of recombinant human secretory leukocyte protease inhibitor (rSLPI) (Stolk et al., 1994). Baicalein and baicalin are extracted from *Scutellaria baicalensis*, and baicalein confers protection in rats against LPS-induced ALI. It inhibits NF-κB-regulated inflammatory responses and upregulates the Nrf2/HO-1 pathway (Tsai et al., 2014). Whereas, in cigarette smoke-induced inflammatory models in mice and A549 cells, baicalin possesses anti-inflammatory properties, which may be mediated *via* decreasing phosphorylation of histone deacetylase 2 (HDAC2) (Li et al., 2012). Moreover, by modulating the Nrf2 pathway and reducing the production of inflammatory cytokines in macrophages, eriodictyol extracted from *Dracocephalum rupestre* was able to reduce LPS-mediated ALI in mice (Zhu et al., 2015). Fisetin, a flavonoid, dramatically lowered lung myeloperoxidase levels as well as the expression of several inflammatory mediator genes such as IL-6, TNF-alpha, IL-1beta, MIP-1, and MIP-2. Furthermore, fisetin substantially decreased LPS-regulated gene expression of HO-1 and SOD2 (Geraets et al., 2009).

Some coumarin compounds such as columbianadin and imperatorin also have anti-inflammatory properties in the lungs (Sun et al., 2012; Lim et al., 2014). Esculin reduced LPS-induced ALI by blocking the activation of MyD88 (myeloid differentiation primary response gene-88). This molecule has been identified to function upstream of NF-κB and NF-κB p65 activation (Tianzhu and Shumin, 2015). Treatment with morin and tectorigenin significantly reduced the number of inflammatory cells in the BALF, and in the lungs, morin lowered the amount of the NLRP3 which is an inflammasome protein. These two constituents also enhanced SOD activity, and downregulated activity of myeloperoxidase (Huang et al., 2013; Tianzhu et al., 2014). Furthermore, zingerone inhibited NF-κB and MAPK signaling pathways by suppressing phosphorylation of p38/MAPK, NF-κB/p65, IκBα, and ERK (Xie et al., 2014).

**FIGURE 3:**
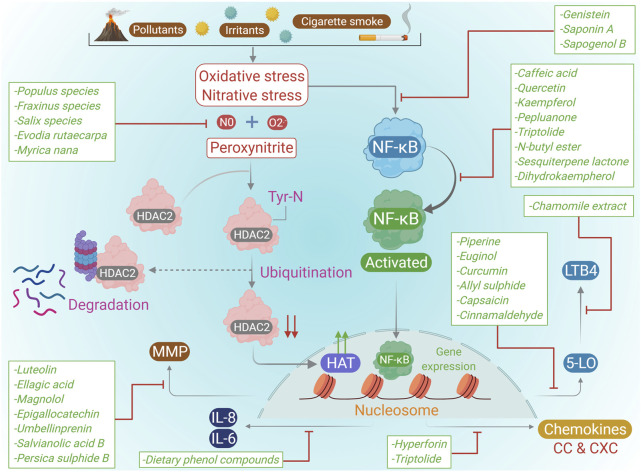
Molecular mechanisms of COPD pathogenesis and the effects of natural plant products.

As stated above, data on various plant components that exhibit inhibitory effects on lung inflammation are constantly growing and some have shown encouraging results. The clinical efficacy of some compounds may be demonstrated in human studies in the near future. Few plant constituents found in different medicinal plants which inhibited lung inflammation are summarized in Table 2. Figure 3 shows the molecular mechanisms of COPD pathogenesis and the effects of plant natural products.

**TABLE 2:** Different plant constituents inhibiting lung inflammation.

Sl. no.	Constituent	Plant origin	Inflammagen used	Class	Reference
1.	Apigenin-7-glucoside	*Andrographis paniculata* (Burm.f.) Nees [Acanthaceae]	LPS (i.t.)	Flavonoid	Li et al. (2015)
2.	Apocynin	*Picrorhiza kurroa* Royle ex Benth. [Plantaginaceae]	LPS (hamster)	Phenol	Stolk et al. (1994)
3.	Baicalein	*Scutellaria baicalensis* Georgi [Lamiaceae]	LPS (i.t.) (rat)	Flavonoid	Tsai et al. (2014)
4.	Baicalin		Cigarette smoke	Flavonoid	Li et al. (2012)
5.	Berberine	-	Cigarette smoke	Alkaloid	Xu et al. (2015)
6.	Caffeic acid phenethyl ester	Honey-bee propolis	Cigarette smoke (rabbit)	Phenol	Sezer et al. (2007)
7.	Ellagic Acid	-	Acid	Phenol	Cornélio Favarin et al. (2013)
8.	Eriodictyol	*Dracocephalum rupestre* Hance [Lamiaceae]	LPS	Flavonoid	Zhu et al. (2015)
9.	Esculin	-	LPS (i.t.)	Coumarin	Tianzhu and Shumin, (2015)
10.	Flavone, Fisetin	-	LPS (i.t.)	Flavonoid	Geraets et al. (2009)
11.	Hesperidin	-	LPS (i.t.)	Flavonoid	Yeh et al. (2007)
12.	Imperatorin	-	LPS	Coumarin	Sun et al. (2012)
13.	Luteolin	*Lonicera japonica* Thunb. [Caprifoliaceae]	LPS (i.t.)	Flavonoid	Lee et al. (2010)
14.	Morin	-	LPS	Flavonoid	Tianzhu et al. (2014)
15.	Naringin	Prunus persica (L.) Batsch [Rosaceae]	Cigarette smoke (rat)	Flavonoid	Nie et al. (2012)
16.	Paeonol	*Paeonia* × *suffruticosa* Andrews [Paeoniaceae]	Cigarette smoke	Phenol	Liu et al. (2014)
17.	Platycodin D	*Platycodon grandiflorum* (Jacq.) A.DC. [Campanulaceae]	LPS (i.t.)	Triterpenoid saponin	Tao et al. (2015)
18.	Sakuranetin	*Baccharis retusa* DC. [Asteraceae]	Elastase-induced emphysema	Flavonoid	Taguchi et al. (2015)
19.	Tectorigenin	*Taraxacum campylodes* G.E.Haglund (= *T. officinale* (L.) Weber ex F.H.Wigg.) [Asteraceae]	LPS	Flavonoid	Huang et al. (2013)
20.	Zingerone	Lichen species	LPS	Phenol	Xie et al. (2014)

## 10 Conclusion

COPD is a serious disease and the conventional treatments are either ineffective or insufficient. Medicinal plants are an important resource for alternative medicine, and numerous powerful medicines have been developed from plants for a variety of human diseases, including respiratory infections. Several plant extracts have the potential to be therapeutically helpful against lung inflammatory diseases such as COPD. Furthermore, several other types of plant components have been shown to suppress inflammatory reactions in the lung. Other plants with relaxing, bronchodilatory, antitussive, anticholinergic, mucociliary clearance, and antispasmodic characteristics might be explored in addition to these. In the future, other cellular pathways will need to be studied to determine the efficacy of natural compounds. Sirtuins, for example, have recently been identified as target molecules in COPD diseases. MMPs are also involved in the regulation of lung elasticity. With continued research, several plant extracts and components may be produced as new disease-modifying drugs for lung inflammatory diseases. In addition to the aforementioned points of view, several safety concerns should be thoroughly explored and investigated. Furthermore, critical studies must first be conducted in animal models to examine the functioning of essential organs and diagnostic markers.

## Glossary

AAIL: azadirachta indica leaf extract
AI: airway inflammation
AKP: alkaline phosphatase
ALB: albumen
A549 cells: basal adenocarcinoma human alveolar epithelial cells
BALF: bronchoalveolar lavage fluid
CJT: Callicarpa japonica Thunb.
COPD: chronic obstructive pulmonary disease
CS: cigarette smoking
CSC: cigarette smoke condensate
EGFR: epidermal growth factor receptor
ERK: extracellular signal-regulated kinases
FTIR: fourier transform infrared spectroscopy
GBD: global burden of disease
HASMC: human airway smooth muscle cells
HDAC2: histone deacetylase 2
IL-6: interleukin 6
JNK: c-Jun N-terminal kinases
LDH: lactate dehydrogenase
LPS: lipopolysaccharide
MDA: malondialdehyde
MMP-9: matrix metallopeptidase 9
MPO: myeloperoxidase
mRNA: messenger RNA
NF-κB: nuclear factor-kappa B
NO: nitric oxide
ROS: reactive oxygen species
SDI: sociodemographic index
SIRT1: sirtuin 1
SOD: superoxide dismutase
TA: total alkaloids
TAA: total alkaloids of aconitum tanguticum
TNF-alpha: tumor necrosis factor-alpha
WBC: white blood cell
