# Corrigendum: Unravelling the therapeutic potential of botanicals against chronic obstructive pulmonary disease (COPD): molecular insights and future perspectives

**DOI:** 10.3389/fphar.2024.1365790

**Published:** 2024-03-07

**Authors:** Sicon Mitra, Uttpal Anand, Mimosa Ghorai, Balachandar Vellingiri, Niraj Kumar Jha, Tapan Behl, Manoj Kumar, Radha, Mahipal S. Shekhawat, Jarosław Proćków, Abhijit Dey

**Affiliations:** ^1^ Department of Biotechnology, School of Engineering and Technology, Sharda University, Greater Noida, India; ^2^ CytoGene Research & Development LLP, Lucknow, Uttar Pradesh, India; ^3^ Department of Life Sciences, Presidency University, Kolkata, India; ^4^ Human Molecular Cytogenetics and Stem Cell Laboratory, Department of Human Genetics and Molecular Biology, Bharathiar University, Coimbatore, India; ^5^ Department of Pharmacology, Chitkara College of Pharmacy, Chitkara University, Chandigarh, India; ^6^ Chemical and Biochemical Processing Division, ICAR-Central Institute for Research on Cotton Technology, Mumbai, India; ^7^ School of Biological and Environmental Sciences, Shoolini University of Biotechnology and Management Sciences, Solan, India; ^8^ Department of Plant Biology and Biotechnology, Kanchi Mamunivar Government Institute for Postgraduate Studies and Research, Puducherry, India; ^9^ Department of Plant Biology, Institute of Environmental Biology, Wrocław University of Environmental and Life Sciences, Wrocław, Poland

**Keywords:** lungs, inflammation, alternative therapy, medicinal plants, COVID-19, COPD, clinical efficacy, plant-based formulation

In the published article, there was an error in the **Affiliation** information about Uttpal Anand [2]. Instead of “Department of Life Sciences, Ben-Gurion University of the Negev, Beer-Sheva, Israel,” it should be “CytoGene Research & Development LLP, Lucknow, Uttar Pradesh, India.”

As a result of the change in affiliation above, the Conflict of Interest statement must be amended to the following.

